# Bicontinuous Interfacially Jammed Emulsion Gels (bijels) as Media for Enabling Enzymatic Reactive Separation of a Highly Water Insoluble Substrate

**DOI:** 10.1038/s41598-019-42769-8

**Published:** 2019-04-24

**Authors:** Sanghak Cha, Hyun Gyu Lim, Martin F. Haase, Kathleen J. Stebe, Gyoo Yeol Jung, Daeyeon Lee

**Affiliations:** 1 Department of Chemical Engineering, Pohang University of Science and Technology, 77 Cheongam-Ro, Nam-Gu, Pohang, Gyeongbuk 37673 Korea; 2 School of Interdisciplinary Bioscience and Bioengineering, Pohang University of Science and Technology, 77 Cheongam-Ro, Nam-Gu, Pohang, Gyeongbuk 37673 Korea; 3 Department of Chemical Engineering, Rowan University, Henry M. Rowan College of Engineering, Glassboro, NJ 08028 USA; 4 Department of Chemical and Biomolecular Engineering, University of Pennsylvania, Philadelphia, PA 19104 USA

**Keywords:** Chemical engineering, Soft materials

## Abstract

Although enzymes are efficient catalysts capable of converting various substrates into desired products with high specificity under mild conditions, their effectiveness as catalysts is substantially reduced when substrates are poorly water-soluble. In this study, to expedite the enzymatic conversion of a hydrophobic substrate, we use a bicontinuous interfacially jammed emulsion gel (bijel) which provides large interfacial area between two immiscible liquids: oil and water. Using lipase-catalyzed hydrolysis of tributyrin as a model reaction in a batch mode, we show that bijels can be used as media to enable enzymatic reaction. The bijel system gives a four-fold increase in the initial reaction rate in comparison to a stirred biphasic medium. Our results demonstrate that bijels are powerful biphasic reaction media to accelerate enzymatic reactions with various hydrophobic reagents. This work also demonstrates that bijels can potentially be used as reaction media to enable continuous reactive separations.

## Introduction

Enzymes are highly efficient natural catalysts that accelerate various reactions with high activity and (stereo-)specificity under mild reaction conditions, making them attractive catalysts for the industry-scale conversion of chemicals^1,2^. Despite these advantages, application of enzymatic reactions are typically limited to the conversion of water-soluble chemicals^3,4^. When a substrate is hydrophobic and thus insoluble in water, its conversion via enzymatic reaction is significantly reduced because the enzyme is typically water soluble and the substrate is present at a very low concentration in the aqueous phase. To enable catalytic conversion of such a hydrophobic substrate, a biphasic system consisting of water and organic solvent is required^5–7^. However, slow mass transfer due to the limited interfacial area between two immiscible liquid phases significantly limits efficient conversion^8–10^. Although the interfacial area could be increased by energy-intensive emulsification, the addition of surfactants^11^, or the use of sponge phases^12,13^ and microemulsions^14–16^, these approaches may not be favored because enzymes can be undesirably deactivated by shear stress^17–19^ or surfactants, resulting in inefficient conversion^20–22^. Surfactants at the interface also form barriers to interphase mass transfer, reducing the efficacy of the enzymatic process. Particle-stabilized Pickering emulsions offer great potential to enable reactive separation of biphasic reactions using enzymes^23–27^. However, these emulsion-based systems do not offer the possibility of performing reactive separation continuously because the dispersed phase remain isolated, making it challenging to add or remove agents to/from the dispersed phase.

**Figure 1:**
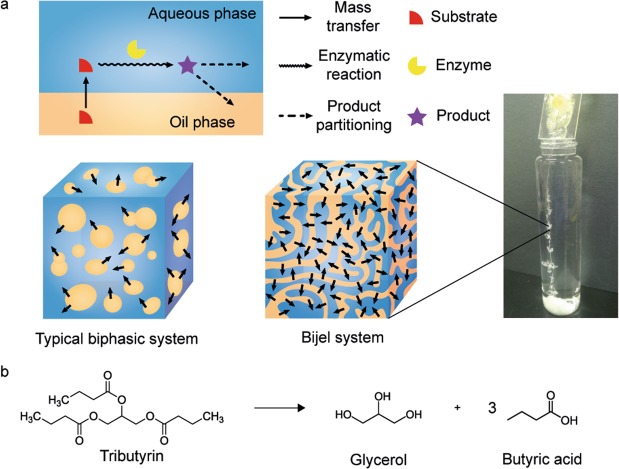
A schematic diagram of overall strategies used in this study. (**a**) In a biphasic system for the enzymatic reaction, the enzyme and the substrate exist in the aqueous phase and the oil phase, respectively. Once the substrate in the oil phase is transferred to the aqueous phase, it is converted to product by enzymatic reaction, which will be partitioned to either aqueous or oil phase. While the limited interfacial area of a typical biphasic system impedes the mass transfer from oil to the aqueous phase, the enormous interfacial area of the bijel system leads to the efficient transfer, facilitating the enzymatic conversion. (**b**) Lipase-catalyzed hydrolysis of tributyrin was chosen as a model reaction.

Recently, a new class of biphasic liquid mixture known as bicontinuous interfacially jammed emulsion gel (bijel) has been introduced. The bijel is a structurally stable biphasic bicontinuous emulsion generated by jamming nanoparticles at the interface between immiscible liquids during spinodal decomposition^28,29^. Unlike typical emulsions, the structures in bijels do not undergo coarsening over time due to the rigidity provided by the interfacially jammed nanoparticle layers. The size of the biphasic domains in bijels can be systematically varied, even below the micrometer scale, by changing the size and concentration of nanoparticles^30^. Although the first examples of bijels were produced by arresting thermally quenched mixtures of oil and water, a recent study has demonstrated that bijels can be continuously produced using a wide variety of oil and nanoparticles by solvent transfer-induced phase separation (STRIPS). This method enables continuous fabrication of bijels in diverse formats (nanoparticle, fiber, and membrane), potentially enabling various applications in biphasic chemical processes (Fig. 1)^30^. STRIPS bijels could potentially be used as reaction media for biphasic enzymatic reactions of hydrophobic substrates by enabling rapid interphase mass transfer.

In this study, we test the feasibility of using a STRIPS bijel to induce enzymatic conversion of a hydrophobic substrate in a batch mode. As a model reaction, we choose tributyrin hydrolysis via lipase which requires intimate contact between aqueous and organic phases due to the low solubility of the substrate in water. This reaction has been widely utilized to assay the kinetic activity of lipase^31–34^. Bijel fibers containing tributyrin in the oil phase are produced with different concentrations of silica nanoparticle and cetyltrimethylammonium bromide (CTAB) to control the domain sizes. We show that the bijel fiber with the smallest domains and thus highest interfacial area leads to the highest rate of enzymatic conversion of tributyrin. When compared to a conventional biphasic system that has been used to induce conversion of hydrophobic substrates, the bijel system gives almost four-fold increase in the initial reaction rate, demonstrating that the bijel can potentially be used as a biphasic medium for the continuous enzymatic conversion of hydrophobic substances.

## Results

### Fabrication of bijel fiber containing tributyrin

**Figure 2:**
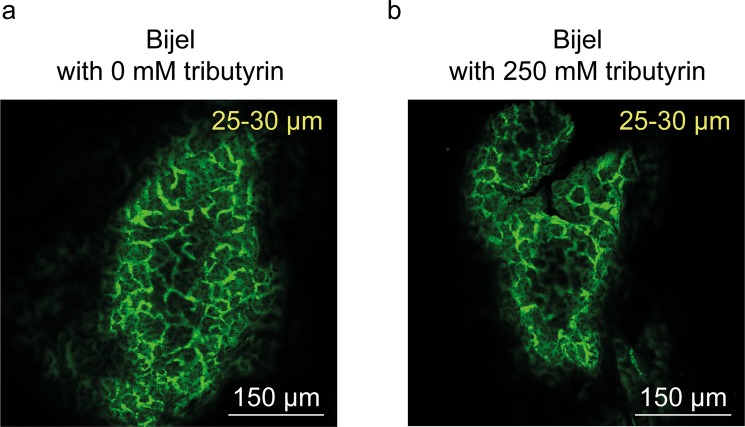
Confocal images of bijel fibers (**a**) without tributyrin and (**b**) with tributyrin. The oil phase of bijel was visualized by addition of the hydrophobic fluorescent dye (Nile Red) in the oil phase. The numbers indicate the pore size of each bijel fiber. The scale bar represents 150 μm.

The fabrication of bijel fiber by the STRIPS method uses a homogeneous ternary mixture consisting of two immiscible liquids (water and diethylphthalate) and a co-solvent, ethanol. To employ the STRIPS bijel fiber for the conversion of tributyrin (Fig. 1), we added this substrate in the oil phase. We also used 2.4 wt% SiO_2_ nanoparticle and 53 mM CTAB for stabilization of the bijel, similar to a previous study^28^. To ensure that the addition of the substrate did not alter bijel formation, we confirmed its morphology via confocal microscopy. As shown in Fig. 2, we observed typical bijel structures, regardless of addition of tributyrin. The domain sizes were approximately 25–30 μm regardless of addition of tributyrin, indicating successful formation of bicontinuous biphasic media with large interfacial area for enzymatic reaction.

### Effect of SiO_2_ and CTAB concentration to the domain size of bijel fiber

When spinodal decomposition is triggered via STRIPS, silica nanoparticles, modified *in situ* by CTAB molecules, attach to the oil-water interface and undergo jamming to arrest the phase separation and thus stabilize the resulting bicontinuous microstructures. Since the point at which interface becomes jammed is determined by the amount of nanoparticles present in the ternary mixture, the concentrations of SiO_2_ nanoparticle and CTAB are critical factors that determine the domain size of the fabricated bijel fiber^30,35^. At elevated nanoparticle concentrations with sufficient surfactant, interfacial jamming arrests the phase separation in the early stages and thus stabilizes small structures, resulting in small domain size. In contrast, the domain size would be increased if nanoparticles do not attach to the interface efficiently due to the lack of CTAB that modifies the nanoparticles.

**Figure 3:**
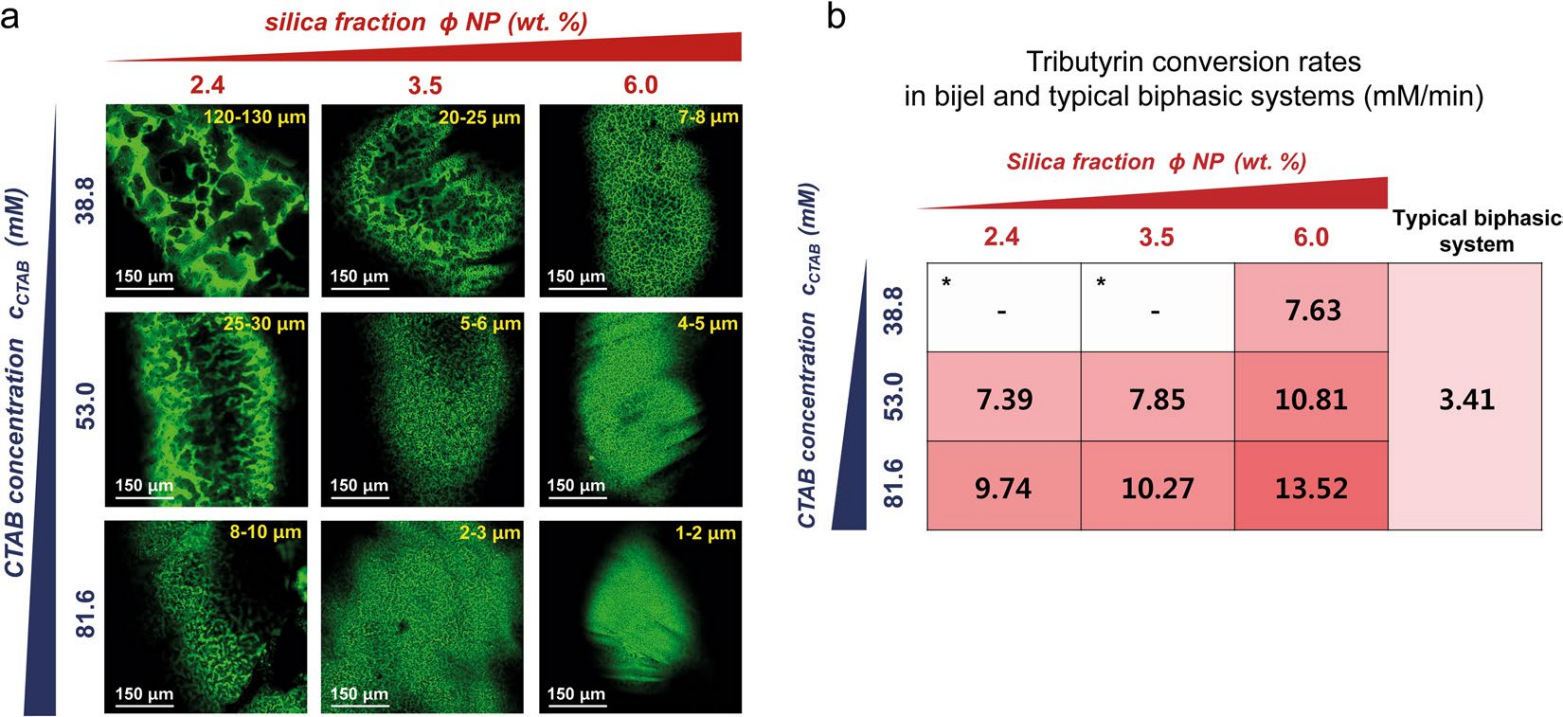
Effect of concentration of stabilizing reagents (SiO_2_ nanoparticle and CTAB) to pore size and the reaction rate of tributyrin hydrolysis. (**a**) Confocal images of different bijel fibers prepared with various concentration of SiO_2_ nanoparticle (2.4, 3.5, 6.0 wt%) and CTAB (38.8, 53.0, 81.6 mM). The oil phase of the bijel was visualized by addition of the hydrophobic fluorescent dye (Nile Red) in the oil phase. The numbers indicate the pore size of each bijel fiber. The scale bar represents 150 μm. (**b**) Comparison of tributyrin conversion rate (mM/min) between the bijel system and the conventional biphasic system.

To control the domain size of STRIPS bijel fibers, we varied the concentration of SiO_2_ nanoparticles (2.4, 3.5, 6.0 wt %) and CTAB (38.8, 53.0, 81.6 mM) in the quaternary mixture. As expected, we observed that the domain size in the bijel fibers changed by 100-fold (from 130 μm to 1 μm) by changing the concentration of SiO_2_ nanoparticle and CTAB (Fig. 3a). The domain size decreased with increased concentration of the SiO_2_ nanoparticle and increased CTAB concentration. With 6.0 wt% SiO_2_ nanoparticle and 81.6 mM CTAB, we successfully obtained bijel fibers with domain sizes ranging between 1 and 2 μm.

### Enhanced enzymatic conversion of tributyrin with bijel fiber

To investigate the effect of the interfacial area of bijel fiber, we compared the initial reaction rates of tributyrin hydrolysis with the bijel fibers with different domain sizes. Two bijels that were prepared with 2.4 or 3.5 wt% nanoparticles along with 38.8 mM CTAB were unstable during buffer exchange. We focused on conducting enzymatic hydrolysis with the remaining bijel fibers.

In this lipase reaction, one mole of tributyrin is converted to one mole of glycerol and three moles of butyric acid (Fig. 1b). Therefore, we quantified the concentration of butyric acid in the aqueous phase upon the termination of the enzymatic reaction. As illustrated in Fig. 3b, the reaction rates generally increased with a decrease in the domain size, indicating that large interfacial area of bijel is critical to improve the rate of the enzymatic reaction. In particular, the bijel fiber with the smallest domain size showed close to a two-fold increase in the reaction rate (13.52 mM/min) than that with the largest domain size (7.39 mM/min). We also compared this bijel system to the conventional biphasic system which was formed by agitating an oil/water mixture at 150 rpm using an orbital shaker. Similar methods have been used previously to induce enzymatic conversion of hydrophobic substrates in biphasic mixtures^36–38^. The reaction rate in the bijel system showed close to four-fold enhancement compared to the conventional biphasic reaction system. This result demonstrates that bijels with large interfacial area function as an effective reaction medium to accelerate the enzymatic conversion of poorly water-soluble substances. To the best of our knowledge, this is the first report on demonstrating the application of bijel for enzymatic conversion of hydrophobic substrates in a biphasic medium.

## Discussion

The main bottleneck during enzymatic conversion of a water-insoluble substrate in a biphasic medium is the limited interphase mass transfer to the enzyme-containing aqueous phase^39,40^ due to limited interfacial area. In this study, we have demonstrated that bijel prepared by the STRIPS method can be effectively used as a biphasic reaction medium with high interfacial area to enhance the enzymatic hydrolysis of tributyrin. Because of the versatility in preparing bijels with various sets of nanoparticles and oils, STRIPS bijels could potentially be used in various biphasic enzymatic reactions with various hydrophobic and highly water insoluble substrates. Additionally, other versatile bijels for the conversion of poorly water-soluble substances can potentially be developed to exploit nanoparticles with catalytic activities^41,42^.

It is important to place our current work in the context of bijel development since it was first described in 2005. A key envisioned application was to use bijels as media to enable continuous reactive separations. The results presented in this work, albeit in a batch mode, clearly demonstrate that STRIPS bijels can be used as reaction medium to induce reactive separation involving enzymatic conversion of hydrophobic substrates and interphase mass transfer of chemicals. Several additional hurdles, however, must be cleared to fully realize the original vision of continuous reactive separation using bijels. For example, mass transfer of the reactant and/or product in the two liquid phases could potentially limit the efficiency of reactive separation. One potential approach to overcome such a challenge is to induce convection in one or both liquid phases. A recent development has shown that bijels can be reinforced to withstand shear and flow^43^. By combining these recent advances in the preparation and application of bijels, we believe our current work brings us one step closer to realizing the vision of bijel-based continuous reactive separations.

## Methods

### Chemical reagents and apparatus

Most chemical reagents were purchased from Sigma (St. Louis, MO, USA) unless otherwise mentioned. For bijel fabrication, Ludox TMA colloidal silica (SiO_2_, 34 wt. % suspension in water), cetyltrimethylammonium bromide (CTAB, BioUltra > 99%), diethylphthalate (DEP, 99.5%), absolute ethanol (analytical grade), Nile Red, and tributyrin (97%, FG) were used. Lipase from porcine pancreas (Type II, Sigma catalog number L3126) was used for conversion of tributyrin. Round glass capillary (outer diameter 1.0 mm, inner diameter 0.58 mm) and square capillary (outer diameter 1.5 mm, inner diameter 1.05 mm, length 150 mm) were obtained from World Precision Instruments (Sarasota, FL, USA) and Atlantic International Technologies Incorporation (Rockaway, NJ, USA), respectively. The diameter of tip for the round glass capillary was narrowed to be 20–300 μm. The round capillary was inserted into the square capillary and aligned concentrically to fabricate the device for STRIPS bijel fabrication. The capillaries were coated with polydiallydimethylammonium chloride (PDADMAC) to avoid undesirable adsorption of extruded bijel fibers. The syringe pumps were purchased from KD Scientific (Holliston, MA, USA).

### Fabrication of bijel fiber by the STRIPS method

Bijel fibers were prepared by the STRIPS method as previously reported^30^. Specifically, bijel fibers were fabricated by injecting ternary mixture and continuous phase solutions through round and square glass capillaries, respectively. The extruded ternary mixture was collected in a container filled with the continuous phase solution.

The ternary mixture was prepared by mixing 5 different liquids: (i) de-ionized water (ii) SiO_2_ nanoparticle suspension in water at pH 3 (pH was adjusted by addition of 1 M HCl for uniform dispersion of silica and the concentration was changed to 32.4 wt% during pH adjustment) (iii) absolute ethanol (iv) CTAB solution (200 mM CTAB dissolved in ethanol) (v) DEP. The final 1 mL (as an example volume) of the mixture contains 0.076 mL of water, 0.108 mL of SiO_2_ nanoparticle suspension, 0.143 mL of absolute ethanol, 0.265 mL of CTAB solution, and 0.408 mL of DEP. This ternary mixture contains 41 vol% DEP, 41 vol% ethanol, 18 vol% water after mixing. The continuous phase is aqueous solution at pH 3 which contains 1 mM CTAB and 5 wt% ethanol. For the fabrication of bijel fiber for tributyrin conversion, tributyrin-DEP solution (250 mM tributyrin dissolved in DEP) was used instead of pure DEP to result in a quaternary mixture. After fabrication of bijel fiber, the bicontinuous structures were imaged by using a confocal microscope (laser excitation at 480 nm and emission at 600–700 nm) with Nile red staining.

### Enzymatic hydrolysis of tributyrin with bijel and biphasic system

For tributyrin conversion in the bijel system, bijel fiber was fabricated with 2.5 mL of the quaternary mixture containing 1 mL of tributyrin-DEP solution. Upon bijel formation, the continuous phase in the container was completely removed and re-filled with up to 9 mL of 100 mM potassium phosphate buffer (pH 7). This exchange ensured that concentration of CTAB in the reaction solution was reduced below its critical micelle concentration (~1 mM in water)^44^. For a control experiment, 1 mL of tributyrin-DEP solution was added to 8 mL of the buffer to catalyze the reaction under the biphasic system. It should be noted that the amounts of tributyrin are the same in both experiments.

The enzymatic reactions were initiated by adding 1 mL of enzyme solution (250 U/mL lipase in the 100 mM potassium phosphate buffer) and carried out in an orbital shaker (150 rpm) at 37 °C. The experiments were conducted in duplicate. The samples were obtained every 90 seconds and incubated at 65 °C for 20 minutes to deactivate the enzyme. Thereafter, the samples were stored at −80 °C before analysis.

### Analytical methods

The enzymatic reaction rate was calculated based on the amount of produced butyric acid. To measure the amount of butyric acid, we quantified its concentration by high-performance liquid chromatography (HPLC). Subsequently, we estimated the butyric acid in oil DEP by considering the partitioning coefficient of the butyric acid in DEP and water.

For HPLC analysis, UltiMate^TM^ 3000 analytical HPLC system (Dionex, Sunnyvale, CA, USA) equipped with an Aminex HPX-87H column (Bio-Rad Laboratories, Richmond, CA, USA) was used. As the mobile phase, 5 mM of H_2_SO_4_ was used at a flow rate of 0.6 mL/min. The temperature of the column oven was set to 65 °C. The refractive index (RI) signal was monitored by a Shodex RI-101 detector (Shodex, Klokkerfaldet, Denmark).
